# Influence of vegetation structure, seasonality, and soil properties on rodent diversi community assemblages in west Mount Kilimanjaro, Tanzania

**DOI:** 10.1002/ece3.9211

**Published:** 2022-09-19

**Authors:** Suzana M. Thomas, Geofrey E. Soka, Loth S. Mulungu

**Affiliations:** ^1^ The African Centre of Excellence for Innovative Rodent Pest Management and Biosensor Technology Development Project Sokoine University of Agriculture Morogoro Tanzania; ^2^ Department of Wildlife Management Sokoine University of Agriculture Morogoro Tanzania; ^3^ Department of Biology University of Dodoma Dodoma Tanzania; ^4^ Institute of Pest Management, Sokoine University of Agriculture Morogoro Tanzania

**Keywords:** community assemblage, Mount Kilimanjaro, rodent diversity, vegetation structure

## Abstract

Rodent diversity and community assemblages are affected by several biotic and abiotic factors such as vegetation structure and seasonality. Vegetation structure particularly ground cover influences rodent diversity and community assemblages through provision of food resources and protection from predators. Such information is important for understanding species–habitat relationships for management and conservation. This study was conducted to determine the influence of vegetation structure, seasonality, and soil properties on species richness, abundance, community assemblages, and habitat association of rodents in west Mt Kilimanjaro. Rodent trapping was conducted using removal and capture–mark–recapture (CMR) methods with medium‐sized Sherman's live traps, snap, and Havarhart traps. Rodents were trapped during wet and dry seasons for three consecutive nights at 4 weeks intervals from April 2020 to March 2021. Environmental variables including vegetation structure, soil physical properties, and disturbance levels were recorded for each habitat type. Fourteen species of rodents were trapped in 25,956 trap nights. *Rhabdomys pumilio*, *Praomys delectorum*, and *Lophuromys verhageni* were the most dominant species across all habitats and seasons. *L.verhageni* occurred in all habitats while *R.pumilio* was restricted from occurring in montane forests. Moreover, species richness and abundance were influenced by habitat types, seasonality, soil type, and ground cover. Generally, both species richness and abundance were higher in fallows and montane forests and significantly lower in plantation forest and agricultural fields. In addition, rodent diversity was highest in fallows, followed by montane forests, and lowest in agricultural fields. Furthermore, rodents were associated with habitat types and vegetation structure forming two major community assemblages that significantly differed between habitats. Our study conclude that, community assemblages of rodents on Mt. Kilimanjaro were affected by functional spatial heterogeneity of the habitats occupied. Therefore, use of different habitats by rodents may be indicative of the landscape integrity and ecosystem changes based on species assemblages.

## Graphical abstract

We used Capture Mark Recapture (CMR) and Removal methods to investigate the influence of vegetation structure, seasonality and soil physical properties on rodent community assemblages. Results indicated that rodent species richness and abundance were influenced by the variation in vegetation structure, seasonality and soil texture. Moreover, vegetation structure was the most important factor influencing rodent community assemblages along the altitudinal gradient.

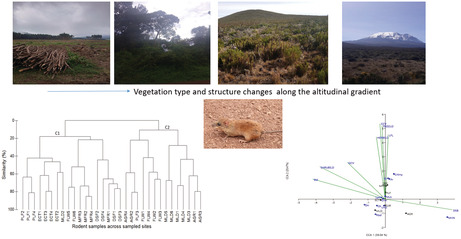

## 1 INTRODUCTION

Rodents are among the most diverse and widely distributed mammals on earth. This is due to their ability to inhabit natural and seminatural habitats and consume almost everything (Kay & Hoekstra, 2008).They play a great role in ecological systems such as pollination and seed dispersal (Johnson et al., 2011). Rodents have low movement patterns and small home ranges (Saanya et al., 2021), which make them sensitive to changes in vegetation structure at smaller scales (Malcolm & Ray, 2000; Stirnemann et al., 2015); hence, they serve as ecological indicators of the environment (Avenant, 2003, 2011). The influence of habitat types, vegetation structure, and composition on rodent diversity and community assemblages is underlined by the habitat heterogeneity hypothesis (Stevens & Tello, 2011). The habitat heterogeneity hypothesis explains that heterogeneous habitats support high species diversity due to increased microhabitats that provide more niches for coexisting species (August, 1983; Stein & Kreft, 2015). Heterogeneous habitats or habitat patches affect rodent diversity, abundance, and community assemblages through the provision of alternative microhabitats that serve as refuges and provide limiting resources to habitat generalists (Cramer & Willig, 2002; Mayamba et al., 2019, 2020; Stein & Kreft, 2015). The influence of vegetation structure has been a central focus in the community ecology of small mammals including rodents (Cramer & Willig, 2002). Vegetation structure is among the most determinant factors of rodent species diversity, composition, and abundance (Admas & Yihune, 2016; Bantihun & Bekele, 2015; Chidodo et al., 2020; Cramer & Willig, 2002; Grelle, 2003; Sullivan et al., 2000; Torre Corominas, 2004). Generally, the influence of vegetation structure on rodent community is determined through habitat associations (Admas & Yihune, 2016; Bantihun & Bekele, 2015; Chidodo et al., 2020; Cramer & Willig, 2002).

In addition, rodent diversity and community assemblage are influenced by many factors such as food availability, competition, predation, diseases and parasites, soil properties, climate, and altitude (Torre Corominas, 2004). For example, seasonal variations in rainfall distribution affect food quantity and quality which influences rodent's diet (Mulungu et al., 2011) and breeding patterns (Leirs et al., 1994, 1997; Makundi et al., 2005, 2007; Mulungu et al., 2013). Physical properties of soil such as soil type/texture, bulk density and soil moisture influences the distribution, population size and survival of rodents due to burrowing for nests and cover (Massawe et al., 2008; Mlyashimbi et al., 2019). Furthermore, elevation range influences rodent species composition and distribution through vegetation zoning. Also, climate variability and anthropogenic activities in low altitudes affect vegetation zoning and rodent species distribution (Hemp, 2006; Lema & Magige, 2018; Mbugua, 2002).

Mount Kilimanjaro is the highest mountain in Africa (roof of Africa) and the world's famous heritage site and tourist attraction, with high diversity of rare and endemic small mammals including rodents (Grimshaw et al., 1995; Shore & Garbett, 1991; Verheyen et al., 2007). Despite that, research on community ecology of rodents on Mt Kilimanjaro has received relatively little scientific attention than high mountains of East and Central Africa, including Mount Elgon in Kenya and Uganda (Clausnitzer et al., 2003; Clausnitzer & Kityo, 2001), Mount Gecoche in Ethiopia (Bantihun & Bekele, 2015; Yihune & Bekele, 2012), and the Eastern Arc Mountains (Ademola et al., 2021; Chidodo et al., 2020; Makundi et al., 2007: Stanley et al., 1998; Stanley & Hutterer, 2007). Most studies on these mountains including Mt Kilimanjaro have been focused on diversity and distribution of rodents along the altitudinal gradients. Previous studies along the Marangu, Mweka, and Shira routes of Mt. Kilimanjaro provided checklists and the distribution of rodent species in association with altitude (Grimshaw et al., 1995; Grimshaw & Foley, 1991; Mulungu et al., 2008; Stanley et al., 2014). However, none of these studies investigated the influence of vegetation structure, seasonality, and soil properties on rodent community assemblages. Such knowledge is relevant to park managers for understanding species–habitat relationships for management and conservation purposes. Therefore, we aimed to determine the influence of vegetation structure, seasonality, and soil properties on rodent species richness and abundance in west Mt. Kilimanjaro. Second, we aimed to determine community assemblages and habitat association of individual rodent species. We hypothesized that: (H1) Variations in vegetation structure, seasonality, and soil properties affect rodent species richness and abundance. We predict high rodent species richness and abundance in heterogeneous habitats. Heterogeneous habitats have high primary productivity and ground cover which improves food availability and reduce predation risk (Cramer & Willig, 2002). (H2) Rodent community assemblage is influenced by structural complexity and heterogeneity of a habitat in association with other environmental variables. We predict that, community assemblage would vary remarkably across the habitats with respect to variations in vegetation structure and soil properties (Hernández et al., 2005). Moreover, heterogeneous habitats of Mt Kilimanjaro would support higher diversity and strong interactions of rodent communities due to complex ecosystems as compared with simple habitats (Mulungu et al., 2008).

## 2 MATERIAL AND METHODS

### 2.1 Study site description

**FIGURE 1:**
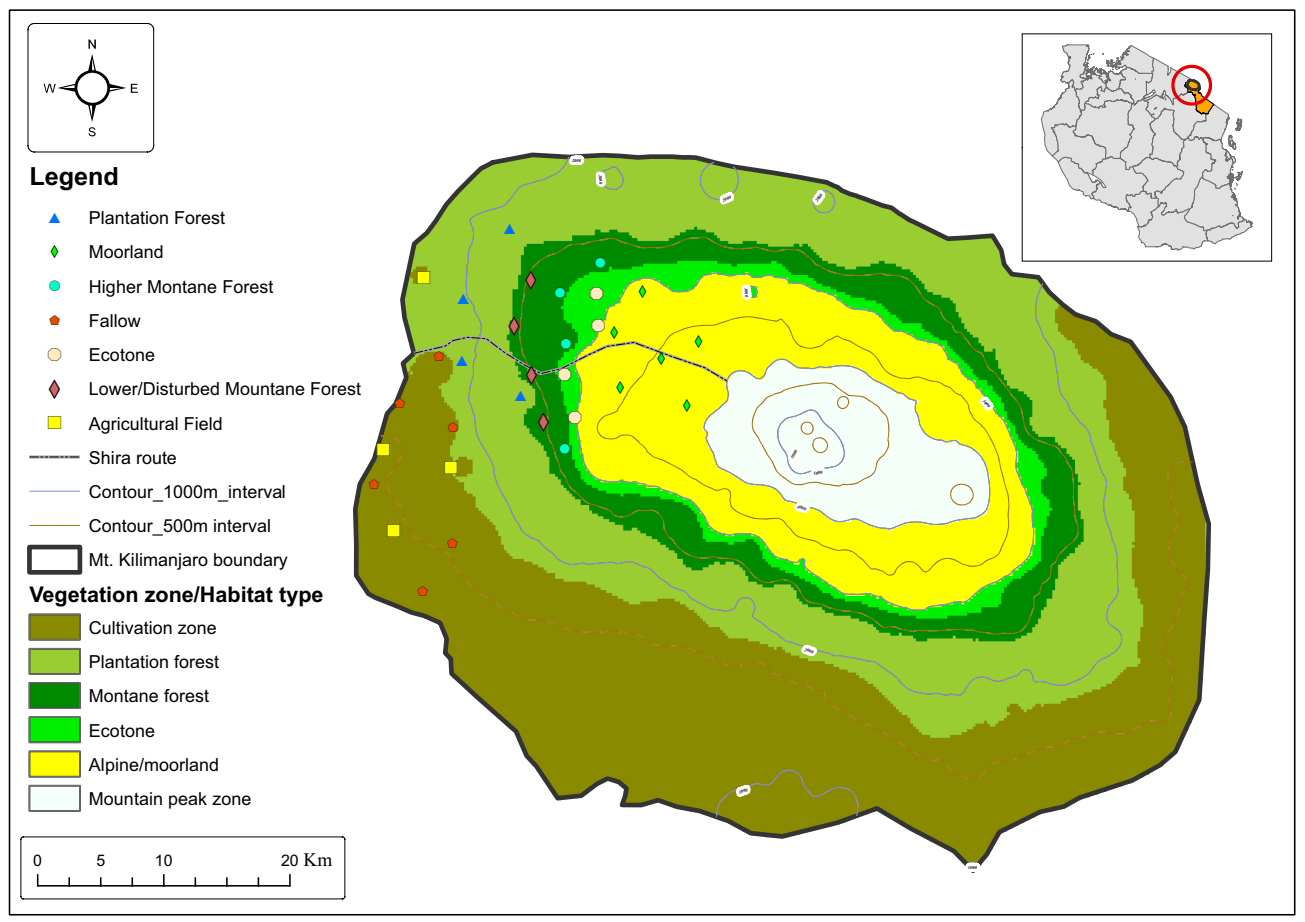
Map of Mt Kilimanjaro showing study sites in the selected habitats along the Shira route (in West Kilimanjaro).

The study was conducted on Mount Kilimanjaro which is located in northeastern Tanzania. The study area lies between 3°07S and 37°35E on the western slopes of Mt Kilimanjaro in Siha district, covering a total area of 1668 km^2^ and reaching a maximum altitude of 5895 m a.s.l. (Figure 1). According to Mulangu and Kraybill (2013) the Mountain is characterized by a tropical montane climate with two rainy and two dry seasons. Rainy season 1 is a long and major season from March to May, and rainy season 2 is a short and minor one from October to December. Also, there is dry season 1 which is the shortest and driest one from January to February, as well as dry season 2 which is long and less dry from June to September. Frosts are also common from June to August during the nights (Thompson et al., 2002). The estimated mean annual rainfall ranges from 700 mm in the lowlands to around 2200 mm in highlands. The general range of temperatures is between −6°C in the highlands and 29°C in the lowlands. The parent material for most soils in the area is volcanic ash and pumice which are typically well‐drained. The soils are highly fertile and predominantly dark grayish, dark brown, and dark yellowish‐brown with sandy and clay loams (Nanzyo et al., 1993).

Generally, the mountain is covered with a zonation of habitat types along the altitudinal gradient (Hemp, 2006; Mulungu et al., 2008). Habitat types were classified as plantation forest and cultivated zone, montane rain forest, alpine heath, and moorland. Plantation forest and cultivated zones range from 1500 to 2400 m a.s.l. covering a total area of 7630 ha. It occupies a transition zone between human settlements with an estimated human population of 2500 people (Mbonile et al., 2003; National Bureau of Statistics, 2012). This zone includes agricultural fields and farms, fallows, and plantation forests. The latter habitat is comprised of extensive tree stands of *Pinus patula*, *Grevillea robusta*, *Eucalyptus* spp, *Cupressus lusitanica*, and *Acrocarpus fraxinifolius*. Also, within young plantations, there are cultivated agricultural fields under the taungya system, a free space between newly planted trees accommodating seasonal crops mainly carrots (*Daucus carota*), cabbage (*Brassera oleracea*), green peas (*Pisum sativum*), and Irish potatoes (*Solanum tuberosum*).

The montane rain forest zone is found in both WKFR (West Mt. Kilimanjaro Forest Reserve) as a remaining natural forest from human disturbance named lower montane forest (DSF) and largely in Mt. Kilimanjaro National Park KINAPA named higher montane forest (MFR). The montane forests has indigenous tree species such as *Podocarpus latifolius*, *Olea europea*, *Ficus thonningii*, and *Cassipourea molasana*. Others are *Schefflera* spp, *Juniperus procera*, *Hagenia abyssinica*, and *Cussonia spicata*. It is an evergreen rainforest dominating from 1800 m a.s.l. up to 2800 m a.s.l., and the wettest part receiving up to 2300 mm of annual rain fall. Alpine heath or ecotone was another habitat type observed from 2800 to 3200 m a.s.l. transitioning to moorland. In this zone, there is sparser and drier vegetation than in the montane rain forest dominated by *Erica excelsa* and *Philippia trimera* shrubs. The heath/ecotone also includes bearded lichen which hangs from the *Erica excelsa* and other trees mostly *Hagenia abyssinica* and *Podocarpus* spp. The annual rainfall is around 1300 mm and such grasses as *Agrostis producta*, *Festuca convoluta*, and *Koeleria gracilis* dominate this area. Lastly, a subalpine zone with a moorland habitat type was evident from 3200 m a.s.l. dominated by Erica bush and changing to *Helichrysum* spp. up to 4500 m a.s.l. as well as rocky and bare land (Hemp, 2006). *Protea kilimandscharica*, *Kniphofia thomsonii*, and *Lobelia deckenii* are also prevalent. It is the coldest with day and night temperatures ranging from 10 to 21 and −1 to 10°C respectively.

### 2.2 Study design and sampling procedures

The study was purposively conducted in seven habitat types: agricultural fields AGR, fallows FLW, plantation forest PLF, lower DSF and higher MFR montane forests, ecotone/alpine heath ECT, and moorland MLD between April 2020 and March 2021. To maximize capture and diversity of rodents two methods, capture–mark–recapture/release (CMR) and removal techniques were employed for rodent trapping with a combination of different traps as conducted by Welegerima et al. (2020) and Shilereyo et al. (2020).

In capture–mark–recapture (CMR) method, permanent experimental grids of 70 m × 70 m (with a 10 m buffer from the edges) were established in both fallows, higher montane forest, and moorland. Two replicate grids at a minimum distance of 500 m were established in each of the fallow and moorland habitats and three replicate grids in higher montane forest, making a total of seven grids. For each grid, medium‐sized Sherman's live traps (23 × 9.5 × 8 cm H.B. Sherman's Traps, Inc.) were arranged in seven lines with seven trapping stations 10 m apart making a total of 49 traps. Traps were baited with peanut butter mixed with maize flour and left for three consecutive nights. Trapping was conducted every month at a 4‐week interval. Traps were inspected every morning before 10:00 am to avoid death and suffocation from harsh weather conditions. Trapped individuals were toe clipped and coded following animal health and safety marking procedures (Borremans et al., 2015). Animals were weighed, sexed, and their reproductive conditions examined. Finally, trapped animals were released at a capture station, and the traps were rebaited for the next trapping night.

In the removal method, trapping was conducted in all seven habitat types using a combination of traps following procedures described in Shilereyo et al. (2020) and Welegerima et al. *(* 2020). For each habitat type, at least four plots were randomly selected. Five transect lines 50 m long and 10 m apart were established in each plot. Sherman and snap traps (1.0 × 8.5 × 16.5 cm) were alternately placed in 10 trapping stations spaced 5 m apart. In addition, four wire cages/Havahart traps (60 × 15 × 170 cm) were randomly placed in the plot specifically for trapping larger species such as *Cricetomys* and squirrels (Shilereyo et al., 2020; Welegerima et al., 2020). In total, 54 traps (twenty‐five Sherman, twenty‐five snaps and four Havaharts) were employed in each of the plot. Sherman traps were baited with peanut butter mixed with maize flour. Snap traps were baited with coconut and Havahart traps with either bananas, carrots, or roasted meat. The traps were left for three consecutive nights at an interval of 4 weeks. Some of the trapped animals from Sherman traps were euthanized (killed humanly) using Halothene solution soaked in cotton wool so as to remove tissue samples such as muscles, liver, and kidney for further research. The animals were weighed, sexed, and morphometric measurements such as head‐body, tail, and hind leg lengths were recorded. The rest were released at capture site. Larger animals from Havahart traps were anesthetized, had their ears pierced, and released at the capture site. Whereas, animals from snap traps were dissected, and their stomachs preserved in 70% ethanol for further research.

Animals caught using both methods (CMR and removal) were identified to species level following Happold (2013) and Monadjem et al. (2015). Toe clip tissue samples were preserved in 99% ethanol for further molecular identifications. Some species (from the removal method) were collected as voucher specimens that are deposited in the museum at the Institute of Pest Management of Sokoine University of Agriculture, Tanzania.

### 2.3 Habitat characterization

In each of the seven habitats, two main sample plots each measuring 50 m × 20 m were established on the existing plots/grids used for rodent trapping resulting in a total of 14 plots. A nested quadrant approach which is a modified Whittaker method was employed as narrated by Stohlgren et al. (1995). The plots were used for recording trees encountered within and identified to species level. Tree diameter at breast height (DBH) was measured using a caliper, and tree height was estimated by a Suunto hypsometer. For shrubs, two nested plots of 2.0 m × 2.0 m in each of the 50 m × 20 m main plots were used resulting in 28 subplots. All the shrubs were identified to species level, and their numbers were recorded. For grasses and herbs, 56 nested plots each of size 1.0 m × 1.0 m were established, four within each of the 50 m × 20 m main plots. All grasses and herbs were identified and enumerated. Percentage cover was used as an indirect measure of the performance of the species found within the plot using a scale of 0%–100%. Therefore, a single species covering the entire plot was given a score of 100%. Ground cover was estimated as the total percentage cover of grasses in proportion to bare soil using a scale of 0%–100%. Canopy cover was estimated as the percent of a forest area occupied by the vertical projections of tree crowns following procedures described by Avsar and Ayyildiz (2010). In addition, soil composite samples (250 g) and soil cores at 30 cm depth were collected and preserved in zipper bags for laboratory analysis of soil physical properties such as soil type, pH, bulk density, and soil moisture (Gee & Bauder, 1986).

Disturbance levels were assigned subject to observations in the field and were ranged from 1 to 3. Disturbance levels were based on the presence–absence of human activities such as logging, cultivation, and entrepreneurial facilities (restaurants). History of fire occurrences and disturbance from wild animals were also used. In addition, disturbance levels were based on location of the habitat whether inside or outside the park. For example, agricultural fields and plantation forests were assigned disturbance level 3 (highly disturbed) because they were located outside the national park and were predominated by human activities. Lower montane forest and fallow were assigned disturbance level 2 (moderately disturbed) because they had minimal human intervention despite of being located outside the park. Higher montane forest, ecotone, and moorland were located inside the national park hence were assigned disturbance level 1 (less disturbed only by wild animals).

### 2.4 Data analysis

Trapped animals from both the CMR and removal methods were combined. However, to standardize the sample size, recaptured individuals in the CMR method were not considered for estimating rodent abundance. Following methods by Chidodo et al. (2020), Shilereyo et al. (2020) and Welegerima et al. (2020) rodent abundance was treated as total counts of new captures only. Vegan package 2.4–1 (https://CRAN.R‐project.org/package) in R 3.6.2 (R Core Team, 2013) was used to estimate the abundance of rodents in each habitat. Also, species richness and the Shannon–Wiener diversity index of both rodents and plants were estimated (Oksanen et al., 2013). H′ = −∑pilnpi was used to calculate the Shannon–Wiener diversity index (H′).Where H′ denotes the diversity index and Pi denotes the proportion of individuals found in the ith species (Shannon & Weaver, 1949). Chi‐square test χ^2^ was used to compare the variation in rodent species composition across habitats and seasons. However, following a modified technique by Chidodo et al. (2020), three species such as *Arvicanthis niloticus*, *Pelomys fallax*, and A*ethomys kaiseri* were excluded from the analysis due to their low representation (Table 1). In addition to that, soil samples were processed and analyzed in the laboratory following procedures explained in Gee and Bauder (1986) and FAO (2006).

**TABLE 1:** Species composition of rodents in percentages (number in parentheses) across habitats. The codes correspond to abbreviations of scientific names and habitats types

Species	Habitats							
	AGR	DSF	ECT	FLW	MFR	MLD	PLF	Total
Arv	0 (0)	0 (0)	0 (0)	1 (0.19)	0 (0)	0 (0)	0 (0)	1 (0.07)
Crtmy	0 (0)	10 (10.53)	0 (0)	0 (0)	3 (0.78)	0 (0)	0 (0)	13 (0.93)
Dn	0 (0)	0 (0)	1 (1.61)	34 (6.42)	17 (4.42)	22 (12.09)	1 (2.5)	75 (5.38)
Eith	0 (0)	0 (0)	0 (0)	2 (0.38)	0 (0)	0 (0)	0 (0)	2 (0.14)
Grm	0 (0)	7 (7.37)	4 (6.45)	35 (6.6)	10 (2.6)	0 (0)	1 (2.5)	57 (4.09)
Gr	0 (0)	3 (3.16)	2 (3.23)	2 (0.38)	27 (7.01)	0 (0)	0 (0)	34 (2.44)
LmZ	0 (0)	4 (4.21)	0 (0)	26 (4.91)	0 (0)	0 (0)	0 (0)	30 (2.15)
Lph	11 (11.11)	14 (14.74)	23 (37.1)	92 (17.36)	76 (19.74)	28 (15.38)	16 (40)	260 (18.66)
MnN	41 (41.41)	0 (0)	0 (0)	45 (8.49)	0 (0)	0 (0)	1 (2.5)	87 (6.25)
Mus	0 (0)	1 (1.05)	0 (0)	54 (10.19)	34 (8.83)	0 (0)	3 (7.5)	92 (6.6)
Ot	0 (0)	0 (0)	0 (0)	22 (4.15)	5 (1.3)	4 (2.2)	0 (0)	31 (2.23)
Plf	0 (0)	0 (0)	0 (0)	5 (0.94)	0 (0)	0 (0)	0 (0)	5 (0.36)
Pr	1 (1.01)	54 (56.84)	10 (16.13)	24 (4.53)	213 (55.32)	0 (0)	15 (37.5)	317 (22.76)
Rbd	46 (46.46)	2 (2.11)	22 (35.48)	188 (35.47)	0 (0)	128 (70.33)	3 (7.5)	389 (27.93)
Total	99	95	62	530	385	182	40	1393

Abbreviations: Arv, *Arvicanthis niloticus*; Crtmy, *Cricetomys ansorgei*; Dn, *Dendromus* spp; Eith, *Aethomys kaiseri* (Noack, 1887); Grm, *Grammomys dolichurus* (smuts, 1832); Gr, *Graphiurus murinus* (Desmarest, 1822); LmZ, *Lemniscomys striatus*; Lph, *Lophuromys verhegeni* (Verheyen et al., 2007); MnN, *Mastomys natalensis* (Smith, 1834); Mus, *Mus musculoides* (Temminck, 1853); Ot, *Otomys spp*; Plf, *Pelomys fallax* (peters, 1852); Pr, *Praomys delectorum* (Thomas, 1910); Rbd, *Rhabdomys pumilio* (Spamnan, 1784); AGR, agricultural fields; DSF, lower montane forest; ECT, ecotone; FLW, fallow; MFR, higher montane forest; MLD, moorland; PLF, plantation forest.

General linear models (GLM) were fitted to determine the influence of explanatory variables on species richness and abundance of rodents (Smith & Warren, 2019). Independent variables were both categorical and numerical. The numerical independent variables were soil pH, bulk density, soil moisture, ground cover, canopy cover, tree DBH, plant species richness and diversity. Categorical independent variables were habitat types, soil types, and seasonality. Data were pooled and analyzed into two major seasons (dry and wet). Because other seasons were very short, for example, dry season 1 had only 2 months (January and February). Pearson's pairwise correlation analysis in R was conducted for multicollinearity of the independent variables at *r* ≥ .5 (Appendix A). Correlated variables were excluded from the same model (Smith & Warren, 2019). Before statistical analyses, assumptions of general linear models such as normality (using Shapiro test and Q‐Q plots), independence of variance, and heterogeneity were checked (Smith & Warren, 2019; Zuur & Ieno, 2016). Unlike the data for species richness, rodent abundance did not follow the normal distribution and the data were over dispersed. Due to that, negative binomial distribution models (with log link function) were fitted for rodent abundance. We ran different models in which rodent species richness and abundance were allowed to differ between habitat types, seasonality, and soil types. Also, they were allowed to vary with ground cover, herbs density, soil bulk density, and the interactions between them (Appendix B and C). Akaike information criterion (AIC) was used for model selection whereby the one with the lowest AIC was selected as best model that better describe our data (Burnham & Anderson, 2004). An *F*‐test was used for goodness of fit of the model and *R* ^2^ for the explained variation in rodent species richness. Moreover, two‐way anova (*p* ≤ .05) was used to compare estimates of rodent abundance and species richness across habitats and seasons.

For community assemblages and habitat association of rodents, cluster analysis of rodent samples was performed in the PRIMER v6 program (Clarke & Warwick, 2001). Bray–Curtis similarity matrix with a distance measure was used to cluster the samples (Bray & Curtis, 1957). Previously, the data were square‐root transformed to reduce the influence of dominant species (Clarke & Warwick, 2001). The similarity profile test (SIMPROF) was performed to determine genuine clustering and structuring of rodent samples and statistically test the difference between and within the clusters (Clarke & Warwick, 2001). Analysis of similarity (ANOSIM) test was performed for similarity of rodent community assemblages or clusters between pairs of habitats. Analysis was based on 999 times permutations with the sample statistic Global R (0–1) and the significance level of sample statistic (pi) *p* ≤ .05 (Clarke & Warwick, 2001). Furthermore, canonical correspondence analysis (CCA) was performed in PAST Paleontological Statistics software (Hammer et al., 2002) at the correlation coefficient (*r* ≥ .5). An ordination plot showing the association between individual species and habitat attributes was produced (Hammer et al., 2002; McCune et al., 2002).

### 2.5 Ethical considerations

Our research was approved by the Sokoine University of Agriculture SUA postgraduate committee, Tanzania (Ref no: SUA/DPRTC/PFC/D/2019/0002/13). Registered, approved, and provided a research permit (No: 2020‐163‐NA‐2020‐127) to conduct research on rodents by the Tanzania Commission for Science and Technology (COSTECH) in collaboration with Tanzania Wildlife Research Institute (TAWIRI). An entry permit into Mount Kilimanjaro National Park was granted by Tanzania National Parks (TANAPA). Moreover, the research was conducted following guidelines by the American Society of Mammologists (ASM) for appropriate methods of research on wild animals.

## 3 RESULTS

### 3.1 Rodent species composition

A total of 1393 individuals from 14 species of rodents were trapped on 25,956 trap nights. *Rhabdomys pumilio*, *Praomys delectorum*, and *Lophuromys verhegeni* were the most dominant species contributing to 69.35% of the total captures. *P. delectorum* predominated both higher and lower montane forests with 55.32% and 56.84%, respectively (Table 1). Whereas, *Rhabdomys pumilio* predominated the moorland and agricultural fields with 70.33% and 46.46%, respectively. *R.pumilio* was restricted from occurring in montane forests. However, two individuals were unexpectedly trapped in the lower montane forest. On the contrary, *Lophuromys verhegeni* occurred across all habitats and seasons predominantly in ecotone. *Mastomys natalensis* was the fourth dominant species occurring predominantly in agricultural fields. Other species such as *Aethomys kaiseri*, *Arvicanthis niloticus*, and *Pelomys fallax* had the lowest percentage composition of total captures; with 0.14%, 0.07%, and 0.36%, respectively. Moreover, most species occurred across both habitats and seasons (Tables 1 and 2); however, Chi‐square test indicated that percentage composition (occurrence) of only three species varied significantly across habitats and seasons (Table 3). For example, *P. delectorum (*χ^2^ = 200.38, df = 5, *p* < .001), *L.verhegeni* (χ^2^ = 15.03, df = 6, *p* = .02), and *R. pumilio (*χ^2^ = 377.72, df = 5, *p* < .001). The percentage composition of other species did not statistically differ across habitats and seasons (Table 3).

**TABLE 2:** Abundance and species composition of rodents in percentages (number in parentheses) across the two seasons

Species	Season		
	Dry	Wet	Total
*Arvicanthis niloticus*	0 (00)	1 (0.15)	1 (0.07)
*Cricetomys ansorgei*	8 (1.1)	5 (0.75)	13 (0.93)
*Dendromus* spp	43 (5.93)	32 (4.79)	75 (5.38)
*Aethomys kaiseri* (Noack, 1887)	2 (0.28)	0 (0)	2 (0.14)
*Grammomys dolichurus* (Smuts, 1832)	30 (4.14)	27 (4.04)	57 (4.09)
*Graphiurus murinus* (Desmarest, 1822)	17 (2.34)	17 (2.54)	34 (2.44)
Lemniscomys striatus (Linnaeus, 1758)	16 (2.21)	14 (2.1)	30 (2.15)
*Lophuromys verhegeni*	139 (19.17)	121 (18.11)	260 (18.66)
*Mastomys natalensis* (Smith, 1834)	54 (7.45)	33 (4.94)	87 (6.25)
*Mus musculoides* (Temminck, 1853)	56 (7.72)	36 (5.39)	92 (6.60)
*Otomys* spp	22 (3.03)	8 (1.2)	30 (2.15)
*Pelomys fallax* (Peters, 1852)	3 (0.4)	2 (0.3)	5 (0.36)
*Praomys delectorum* (Thomas, 1910)	133 (18.34)	185 (27.69)	318 (22.83)
*Rhabdomys pumilio* (Spamnan, 1784)	202 (27.86)	187 (27.99)	389 (27.93)
Total	725	668	1393 (100)

**TABLE 3:** Results from Chi‐square test on rodent distribution across both habitats and seasons

Species	χ^2^	df	*p*	Critical value
*Arvicanthis niloticus*	3	3	.39	7.81
*Cricetomys ansorgei*	1.31	1	.25	3.84
*Dendromus* spp	2.61	4	.63	9.49
*Aethomys kaiseri* (Noack, 1887)	6	3	.11	12.59
*Grammomys dolichurus* (Smuts, 1832)	5.11	4	.28	9.49
*Graphiurus murinus* (Desmarest, 1822)	4.68	3	.2	7.81
*Lemniscomys striatus*	1.49	1	.22	3.84
*Lophuromys verhegeni*	15.03	6	**.02**	12.59
*Mastomys natalensis* (Smith, 1834)	3.75	2	.15	5.99
*Mus musculoides* (Temminck, 1853)	3.23	3	.36	7.82
*Otomys* spp	0.19	6	1	12.59
*Pelomys fallax* (Peters, 1852)	4	0	NA	5.99
*Praomys delectorum* (Thomas, 1910)	200.38	5	**<.001**	11.07
*Rhabdomys pumilio* (Spamnan, 1784)	377.72	5	**<.001**	11.07

Abbreviations: χ^2^, Chi‐square test statistic; df, degrees of freedom.

Bold indicated: < .001 = significant at 0***.

### 3.2 Rodent species richness and diversity

Rodent species diversity H (Shannon Wiener diversity Index) was highest in fallow FLW habitat (H = 1.92), followed by lower montane forest DSF (H = 1.64), and lowest in agricultural fields AGR (H = 1.06).

From GLM models, rodent species richness was influenced by both habitat types, seasonality, ground cover, and soil type as they were included in the best model (F_11,1396_ = 95.78, *p* = .001, *R* ^2^ = .43). However, the influence of seasonality was not significant (*p* = .632), and species richness did not significantly differ between dry and wet seasons. Species richness differed significantly between habitats. Whereby, it was highest in fallow but not significant (13 species) followed by both montane forests (higher MFR and lower DSF) (each with eight species) and significantly lower in both moorland and agricultural fields (each with four species, *p* < .001). It was significantly highest in clay soil CLY (0.839 ± 0.165, *p* < .001) and lowest in clay loam soil CLYLM (−1.458 ± 0.205, *p* < .001). Moreover, species richness was positively correlated and increased with increasing ground cover (0.051 ± 0.003, *p* < .001) (Table 4).

**TABLE 4:** Summary of best GLM model (from linear regression) that better describes the influence of independent variables (parameters) on rodent species richness representing estimate, standard error, *Z*‐value and *p*‐value

Parameters	Estimate	Std. error	*Z*‐value	*p*‐value
(Intercept)	0.839	0.165	5.092	4.03e‐07***
Habitat:Lower montane forest	−1.425	0.227	−6.266	4.92e‐10***
Habitat: Ecotone	−1.582	0.207	−7.656	3.58e‐14***
Habitat: Fallow	0.35	0.221	−1.585	.113
Habitat: Higher montane forest	−0.348	0.261	−1.333	.183
Habitat: Moorland	−1.546	0.191	−8.075	1.45e‐16***
Habitat: Plantation forest	−1.32	0.181	−7.312	4.42e‐13***
Season: Wet	−0.046	0.083	−0.56.	.576
GCv	0.051	0.003	17.601	<2e‐16***
Soil: Clay loam	−1.458	0.205	−7.108	1.87e‐12***
Soil: Sandy clay loam	−0.447	0.136	−3.28	.001**
Soil: Sandy loam	−0.835	0.15	−5.575	2.97e‐8***

*Note*: Significant codes: 0 ‘***’ 0.001 ‘**’ 0.01 ‘*’ 0.05 ‘.’ 0.1 ‘ ’ 1.

Abbreviation: GCv, ground cover.

### 3.3 Rodent abundance

The GLM model indicated that, rodent abundance was influenced by the variations in habitat type (*p =* .001), seasonality (*p* = .01), soil type (*p* < .001), ground cover (*p* < .001), and soil bulk density; however, the effect of soil bulk density was not significant (*p* = .06). Rodent abundance differed across habitats and seasons. Abundance (Estimate ± SE, *p*‐value) was highest in fallow FLW but not significant (0.151 ± 0.183, *p* = .408) followed by higher montane forest MFR (−0.031 ± 0.206, *p* = .879) and was significantly lowest in plantation forest PLF (−1.475 ± 0.151, *p <* .001). Moreover, rodent abundance differed between seasons whereby it was significantly higher in the dry season (1.222 ± 0.258, *p* < .001) than in wet season (−0.157 ± 0.067, *p =* .019). Moreover, rodent abundance differed between soil types whereby it was significantly highest in clay soil CLY (1.222 ± 0.258, *p* < .001) and lowest in clay loam soil CLYLM (−1.183 ± 0.172, *p* < .001) than in other soil types. In addition, rodent abundance had a significantly linear relationship with ground cover (0.024 ± 0.002, *p* < .001) and a linear relationship with bulk density (Table 5).

**TABLE 5:** Summary of best GLM model (negative binomial) that better describes the influence of independent variables (parameters) on rodent abundance representing estimate, standard error, *Z*‐value and *p*‐value

Parameters	Estimate	Std. error	*Z*‐value	*p*‐value
(Intercept)	1.222	0.258	4.735	2.19e‐06***
Habitat:Lower montane forest	−0.705	0.183	−3.85	.000***
Habitat: Ecotone	−1.119	0.167	−6.694	2.17e‐11***
Habitat: Fallow	0.151	0.183	0.827	.408
Habitat: Higher montane forest	−0.031	0.206	−0.152	.879
Habitat: Moorland	−0.521	0.152	−3.419	.001***
Habitat: Plantation forest	−1.475	0.151	−9.795	<2e‐16***
Season: Wet	−0.157	0.067	−2.345	.019*
GCv	0.024	0.002	10.01	<2e‐16***
Soil: Clay loam	−1.183	0.172	−6.861	6.85e‐12***
Soil: Sandy clay loam	−0.39	0.132	−2.949	.003**
Soil: Sandy loam	−1.176	0.134	−1.312	.19
BD	0.423	0.214	1.975	.058

*Note*: Significant codes: 0 ‘***’ 0.001 ‘**’ 0.01 ‘*’ 0.05 ‘.’ 0.1 ‘ ’ 1.

Abbreviations: GCv, ground cover; BD, bulk density.

### 3.4 Community assemblages and habitat association

**FIGURE 2:**
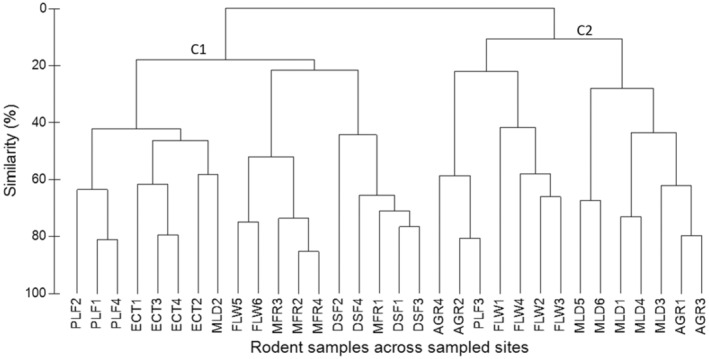
Dendrogram based on Bray–Curtis similarity distance measure showing two broad clusters of rodent communities among the rodent samples across the study area. There was a significant structuring between and within the two major clusters (community assemblages). AGR1‐4, PLF 1‐4, FLW 1‐6, MLD 1‐6, ECT 1‐4, MFR1‐4 and DSF 1‐4 refers to replicated sites in agricultural fields, plantation forest, fallow, moorland, ecotone, higher montane forest, and lower montane forest, respectively.

From cluster analysis based on the Bray–Curtis dissimilarity index, there was evidence of genuine structuring of rodent samples forming two major community assemblages/clusters at 99% efficiency (Figure 2). Community assemblage one (C1) predominated in forested habitats mainly in ecotone, montane (higher and lower), and plantation forests. Whereas, the second community assemblage (C2) predominated in the moorland, fallow, and agricultural fields (Figure 2). The SIMPROF test showed a statistically significant difference between and within the two clusters with sample statistic (pi) of 2.483, *p* = .002 at 999 permutations. Furthermore, the ANOSIM test showed statistically significant differences in community assemblages between pairs of habitats at sample statistic (Global R) = .05, *p* = .01 at 999 permutations (Table 6). For example, agricultural fields AGR were completely distant and significantly different from both lower DSF and higher MFR montane forests (Global‐*R* statistic = 1, *p* = .029) and not significantly different from fallow FLW (Global‐*R* statistic = .218, *p* = .119). Moorland MLD was significantly different from both lower and higher montane forests (Global‐*R* statistic = .833, *p* = .005) (Table 6).

**TABLE 6:** Results from analysis of similarity test ANOSIM on rodent community assemblages at 999 permutations

Pairwise tests					
Groups	*R* statistic	Significance level %	Possible permutations	Actual permutations	Number >=Observed
AGR, DSF	1	2.9	35	35	1
AGR, ECT	.698	2.9	35	35	1
AGR, FLW	.218	11.9	210	210	25
AGR, MFR	1	2.9	35	35	1
AGR, MLD	.333	4.8	210	210	10
AGR, PLF	.625	5.7	35	35	2
DSF, ECT	.698	2.9	35	35	1
DSF, FLW	.349	5.2	210	210	11
DSF, MFR	.51	8.6	35	35	3
DSF, MLD	.833	0.5	210	210	1
DSF, PLF	.37	8.6	35	35	3
ECT, FLW	.262	8.6	210	210	18
ECT, MFR	.792	2.9	35	35	1
ECT, MLD	.143	18.6	210	210	39
ECT, PLF	.188	14.3	35	35	5
FLW, MFR	.508	1.9	210	210	4
FLW, MLD	.435	1.5	462	462	7
FLW, PLF	.361	7.1	210	210	15
MFR, MLD	.833	0.5	210	210	1
MFR, PLF	.49	2.9	35	35	1

*Note*: There were significant differences in rodent community assemblages between pairs of habitats. Sample statistic (global *R*) = .5, significance level statistic *p* = .001.

Abbreviations: AGR, agricultural fields; DSF, disturbed/lower montane forest; ECT, ecotone; FLW, fallow; MFR, higher montane forest; MLD, moorland; PLF, plantation forest.

**FIGURE 3:**
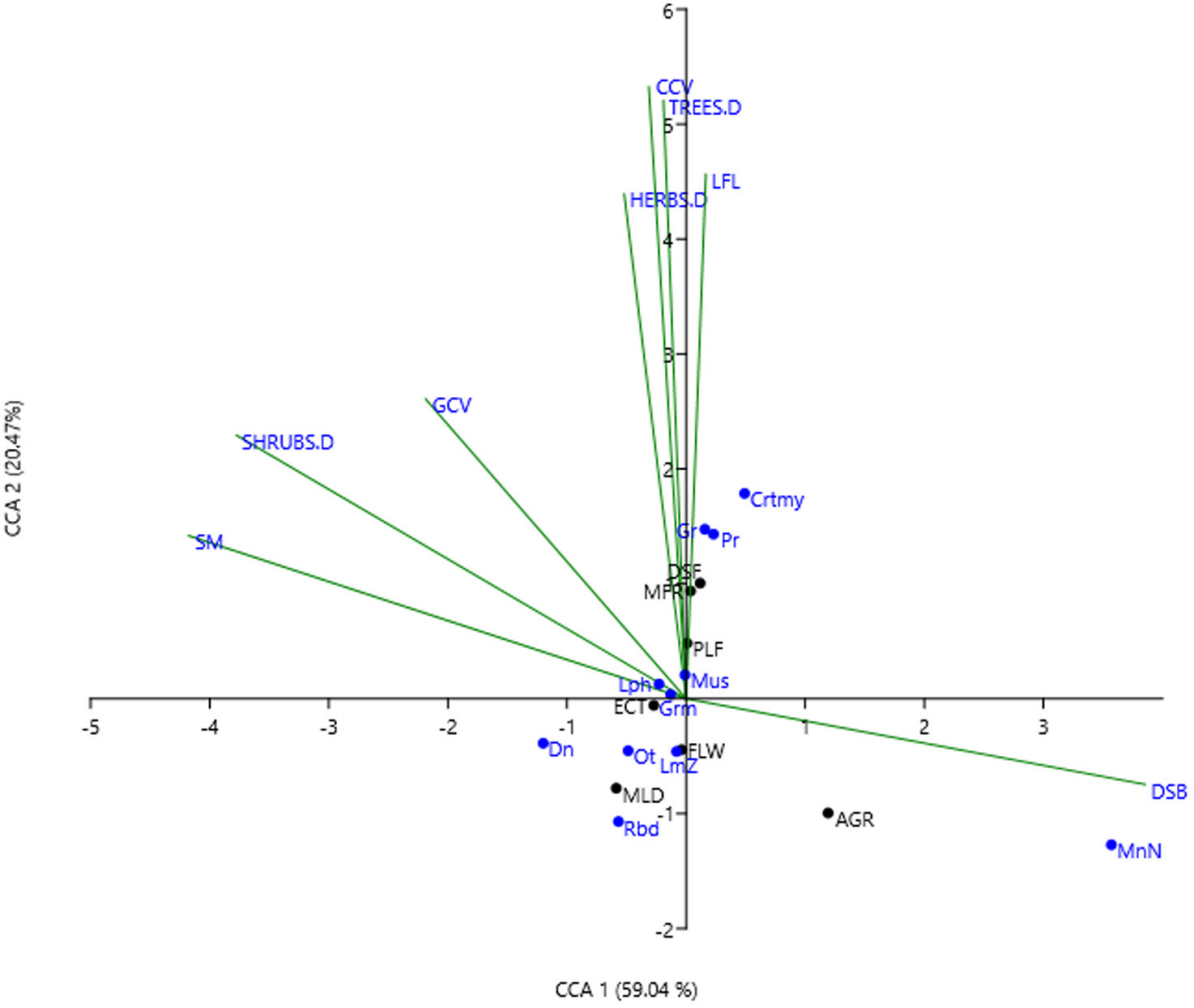
Habitat association of rodents in West Mt Kilimanjaro. Canonical correspondence CCA1explained 59.4% of the variations, while canonical correspondence CCA2 explained 20.47% of the variations. AGR, agricultural fields; DSF, lower montane forest; ECT, ecotone; FLW, fallows; MFR, higher montane forest; MLD, moorland and PLF, plantation forest; LFL, leaf litter; CCV, canopy cover; GCV, ground cover; SM, soil moisture; DSB, disturbance level; Pr, *Praomys delectorum*; MnN, *Mastomys natalensis*; Rbd, *Rhabdomys pumilio*; Ot, *Otomys spp*; Dn, *Dendromus spp*; Gr, *Graphiurus murinus*; Grm, *Grammomys dolichurus*; Mus, *Mus musculoides*; *Lph*, *Lophuromys verhageni*; *LmZ*, *Lemniscomys striatus*; Crtmy, *Cricetomys ansorgei*. SHRUBS.D, TREES.D and HERBS.D = shrub, tree and herb density, respectively.

In addition, CCA canonical correspondence analysis explained about 80% of the variations in two axes (Figure 3). Axis 1 (CCA 1) explained 59.4% of the variation. *Praomys delectorum*, *Graphiurus murinus*, and *Cricetomys ansorgei* loaded positively to canopy cover, leaf litter, tree and herbs density, higher (MFR) and lower (DSF) montane forests. While *R. pumilio* and moorland habitat (MLD) loaded negatively. Indicating that, *P. delectorum*, *G. murinus*, and *C.ansorgei* are more associated with montane forests and their abundance increased with increasing tree and herb density, leaf litter, and canopy cover. While *R. pumilio* was more associated with moorland habitat. Axis 2 (CCA 2) explained 20.47% of the variations with *Dendromus* spp, soil moisture and shrub density loading negatively. While *M. natalensis* loaded positively to disturbance level and agricultural fields. Indicating that, *Dendromus* was more associated with shrub density and soil moisture and their abundance increased with increasing shrub density. Whereas, *M. natalensis* was more associated with agricultural fields and disturbance (Figure 3).

## 4 DISCUSSION

### 4.1 Species composition, community assemblages, and habitat association

Results indicated that, 14 species of rodents were recorded across habitats and seasons. Out of the captured species, two major community assemblages with different composition were formed. Community assemblage one mainly comprised of forest‐adapted species such as *Praomys delectorum*, *Graphiurus murinus*, and *Cricetomys ansorgei*. Whereas, the second community assemblage was mainly comprised of habitat generalists such as *Rhabdomys pumilio*, *Lophuromys verhageni*, *Mastomys natalensis*, *Mus musculoides*, and *Dendromus* spp. The observed community assemblages were probably a result of the variations in vegetation structure across the habitats. Montane forests were characterized by dense and homogenous vegetation which favors forest specialists. Fallow and ecotone were dense and heterogeneous supporting habitat generalists. Whereas, agricultural fields and moorland were homogeneous with sparse vegetation favoring opportunistic species. It is reported that community assemblage of rodents is determined by the coexistence of species which depends on species‐specific traits such as nesting, food availability, and predation risk (Cramer & Willig, 2002). Consistently, in this study, rodents were associated not only with distinct habitat types but also with vegetation attributes. For example, in community assemblage one, *Praomys delectorum* and *Graphiurus murinus* were more dominant in montane forests than in plantation forest. The species were positively associated with tree and herb density, leaf litter, and canopy cover probably because they are habitat specialists and typical forest‐adapted species that prefer areas with dense canopy and vegetation cover. Dense herbs and leaf litter provide enough food, protection from predators, and nesting grounds for the species. Canopy cover maintains humidity and soil moisture which creates suitable microclimate for *P. delectorum* (Bantihun & Bekele, 2015). Similarly, *P. delectorum* has been reported a closed forest dweller that forages on deep leaf litter (Happold, 2013) and builds its nest from litter and other vegetative materials (Monadjem et al., 2015). Moreover, *P. delectorum* has been previously reported as the dominant species in montane forests of Mt Kilimanjaro (Mulungu et al., 2008; Stanley et al., 2014), Mt Elgon in Kenya and Uganda (Clausnitzer et al., 2001) and other mountains including the Eastern Arc Mountains (Ademola et al., 2021; Chidodo et al., 2020; Makundi et al., 2007; Stanley et al., 1998). In addition, *P. delectorum* is reported to inhabit both intact and disturbed forests (Ademola et al., 2021; Gitonga et al., 2016; Monadjem et al., 2015; Mulungu et al., 2008) as well as edges between forest and ecotone (Mulungu et al., 2008). On the contrary, low percentage composition of *P. delectorum* in plantation forest (despite it is a forest‐adapted species) was probably due to high levels of disturbance from anthropogenic activities including cultivation, logging, and firewood collection. These activities result into habitat destruction and fragmentation which adversely affects the survival of native species.

*Rhabdomys pumilio*, *L. verhageni*, *M. natalensis*, and *Dendromus* spp were the most abundant species in the second community assemblage. *R. pumilio* predominated in the moorland and agricultural fields and was moderately associated with ground cover, probably because it is most important and preferred food is grass and seeds hence commonly named the grass rat (Clausnitzer et al., 2001; Shore & Garbett, 1991; Happold, 2013). Moreover, *R. pumilio* occurred in all habitats except in montane forests (however, we unexpectedly caught two individuals in the lower montane forest). This was probably because, *R. pumilio* prefers areas with dry conditions while montane forests of Mt. Kilimanjaro remains wet throughout the year. Consistently, Clausnitzer et al. (2001) reported that *R. pumilio* prefers drier areas with sparse vegetation and bare soil which creates suitable microclimate. Clausnitzer et al. (2001) added that, the species is adapted to cold weathers in the moorland habitat (which gets harsh during the night) by being active during the day. In contrast, Grimshaw et al. (1995) and Stanley et al. (2014) revealed that *R. pumilio* is rarely found in higher montane forests near human habitations with Stanley et al. (2014) trapping few individuals near Horombo tourist huts along the Marangu route of Mt. Kilimanjaro.

Among the captured rodents in this study, *Lophuromys verhageni* was the only endemic species in west Mt Kilimanjaro (Verheyen et al., 2007). It occurred across all habitats and seasons hence termed a habitat generalist. Similarly, species of the same genus have been reported to occur in moist places of montane forests (from 500 m s.a.l in lowland forests) and highland habitats up to 4500 m a.s.l in the Afro‐alpine zone (Bantihun & Bekele, 2015; Happold, 2013). They are widely distributed in bushlands, fallows, plantation forests, montane forests, heath lands, and alpine zones in East, Central, and South Africa (Bantihun & Bekele, 2015; Clausnitzer et al., 2001, 2003; Happold, 2013; Mulungu et al., 2008; Ssuuna et al., 2020; Stanley et al., 1998, 2014; Stanley & Kihaule, 2016).

*Mastomys natalensis* predominated in the agricultural fields (mostly maize, potato, and carrot farms) and was positively associated with disturbance. It was more abundant in the dry season than in wet season. This observation coincides with crop harvest in Kilimanjaro region which is mostly conducted in dry season. Crop remains from harvesting provide supplementary food to *M. natalensis* and other rodents inhabiting the agricultural fields. Similarly, *M. natalensis* is reported as the most common crop pest predominating in agro ecosystems (Mulungu et al., 2013, 2014; Mulungu, 2017). As a habitat generalist and opportunist, *M. natalensis* takes advantage of human disturbance due to the available food resources from cultivation (Happold, 2013; Lema & Magige, 2018; Mulungu et al., 2013, 2014). *Mastomys natalesnsis* together with *Mus musculoides* and *Arvicanthis niloticus* have been reported to prefer agricultural fields and fallows close to human habitation (Admas & Yihune, 2016; Bantihun & Bekele, 2015; Makundi et al., 2010; Mulungu et al., 2006).

*Dendromus* spp were associated with fallow and moorland habitats and positively correlated with shrub density and soil moisture. More individuals of *Dendromus spp* were trapped in dense patches of Erica bushes. Similarly, Happold (2013) reported that *Dendromus* spp is among the species occurring in high abundance above the tree line preferably in dense shrubs and moist places. On the contrary, species such as *Aethomys kaiseri*, *Arvicanthis niloticus*, and *Pelomys fallax* were underrepresented across both habitats and seasons. This observation could be attributed to trapping in higher altitudes from 1500 a m.s.l and above while the species are said to be widely distributed in low‐elevation grasslands and bushes (Grimshaw et al., 1995; Stanley et al., 1998).

Generally, most of the trapped species in this study have been previously captured on both sides of Mt. Kilimanjaro and their distribution and conservation status are well known (Grimshaw et al., 1995; Mulungu et al., 2008; Stanley et al., 2014). However, *Pelomys fallax* has never been previously reported along the Shira route, and therefore, its distribution and conservation status is poorly known. The smaller number of individuals trapped in the current study (*n* = 5) is consistent with Happold (2013) who suggested that *Pelomys fallax* is neither a rare nor an abundant species. Similarly, Mlyashimbi et al. (2019) reported smaller number of *Pellomys fallax* in semiarid areas of Tanzania. However, our results are contrary to Admas and Yihune (2016) who reported similar species of genus Pelomys (*Pelomys harringtoni*) among the most abundant species across habitats of east Gojjam, Ethiopia. Furthermore, in this study, most species have been captured in higher numbers compared with previous studies by Mulungu et al. (2008) and Stanley et al. (2014) in the same study area. This was probably because our study had an extensive sampling period throughout the year covering a relatively large area with a combination of methods and traps.

### 4.2 Influence of vegetation structure, seasonality, and soil type on species richness and abundance

Fallow was the most diverse habitat probably due to high ground cover and shrub density which provide niches for many species (Cramer & Willig, 2002). Fallows are intermediates between agricultural fields and montane forests that serve as refuge to other rodents providing alternative food resources and protection from predators (Cramer & Willig, 2002; Makundi et al., 2010). Montane forests (both higher MFR and lower DSF) were the next diverse habitats with high rodent species richness and abundance. This was probably due to high canopy and ground cover, high vegetation density, and plant species diversity (particularly in the higher montane) forest which provides food and protection to rodents. Lower montane forest on the other hand, had high species diversity and abundance despite the fact that it was less dense than higher montane forest. This was due to moderate disturbance which provided microhabitats to habitat generalists such as *L. verhegeni*, *G. dolichurus* and *Mus musculoides* (Ademola et al., 2021; Mulungu et al., 2008). Similarly, a study by Mulungu et al. (2008) reported maximum rodent abundance in montane forests that decreased above the tree line forming a hump‐shaped distribution, due to maximum rainfall at mid‐elevation (Hemp, 2006). Montane forests receive maximum amount of rainfall which increases primary productivity hence improves vegetation structure and food availability (Clausnitzer & Kityo, 2001). Similar patterns of rodent abundance in montane forests have been reported in the Mabira central forest reserve in Uganda and the Ukaguru Mountains of Tanzania (Ademola et al., 2021; Ssuuna et al., 2020). On the contrary, agricultural fields, plantation forest, and moorland were the least diverse among the seven habitats with lower species richness and abundance. This observation was linked to high disturbance from anthropogenic activities in the agricultural fields and plantation forest which affect the integrity of habitats and reduce diversity of most rodents (Bennett, 1990). In addition, poor vegetation structure and adverse environmental conditions in the moorland affect the survival and distribution of Afro‐alpine rodents (Clausnitzer et al., 2001). Afro alpine environments are characterized by extreme cold weather which restricts movement and activity pattern of rodents forcing them to take cover inside burrows and grasses.

In addition to habitat type and ground cover, seasonality influenced rodent species richness and abundance. However, the influence of seasonality on rodent species richness was not significant probably because most species occurred across both dry and wet seasons. Rodent abundance was relatively higher in the dry season than in wet season. This was probably due to that most species start breeding 1 month after the long rains until the end of wet season. During this period, there is high cover and green foliage which triggers breeding in most rodents (Mlyashimbi et al., 2018). Therefore, rodent population tends to peak 2–4 months later (Mulungu et al., 2013). Similarly, it is reported that the variation in rainfall distribution influence rodents' diet (Mulungu et al., 2011) and breeding patterns (Leirs et al., 1994; Mlyashimbi et al., 2018; Mulungu et al., 2014) through resource availability which in turn affect population abundance (Leirs et al., 1997; Makundi et al., 2007). Moreover, the observed high abundance in dry season could be a result of crop remains in agricultural fields which ensures continuous food supply to rodents inhabiting them.

Furthermore, soil type and microclimate have been reported to influence the distribution, population abundance, and survival of rodents elsewhere (Massawe et al., 2008; Meliyo et al., 2014; Mlyashimbi et al., 2019). In this study, clay soil had higher rodent species richness and abundance than other soils probably because of its good texture. Clay soil hardens during the rainy season allowing the survival of rodents (Meliyo et al., 2014). While other volcanic ash soils of Mt. Kilimanjaro have low bulk density and poor structure that can easily collapse or shrink during rainy season making them unsuitable for most rodents (Nanzyo et al., 1993). However, our results are contrary to those by Mlyashimbi et al. (2019) and Massawe et al. (2008) who reported low abundance and survival of *Mastomys natalensis* and other rodents in clay soils.

## 5 CONCLUSION AND RECOMMENDATION

Results from this study indicated that rodent species richness and abundance in west Mt. Kilimanjaro were a result of several factors including habitat types in synergy with vegetation structure, seasonality, and soil physical properties. Rodent community assemblages reflected the variation in habitat types, vegetation structure, and disturbance level along the altitudinal gradient. Moreover, Mt. Kilimanjaro has heterogeneous habitats that support high diversity of rodents with fallows and montane forests being the most diverse habitats supporting complex communities. However, increasing cultivation and forest plantation in unprotected areas of Mt Kilimanjaro results in habitat destruction and fragmentation. Habitat destruction and fragmentation simplifies vegetation structure favoring the abundance and survival of the habitat generalists and opportunists at the expense of forest‐adapted species. Therefore, the development of ecologically sound strategies is crucial for management and conservation of the rodent communities in Mt Kilimanjaro.

## AUTHOR CONTRIBUTIONS

**Suzana M. Thomas:** Conceptualization (equal); data curation (lead); formal analysis (equal); funding acquisition (lead); investigation (equal); methodology (equal); project administration (lead); resources (equal); software (equal); supervision (supporting); validation (equal); visualization (equal); writing – original draft (lead); writing – review and editing (equal). **Geofrey E. Soka:** Conceptualization (equal); data curation (supporting); formal analysis (equal); funding acquisition (supporting); investigation (equal); methodology (equal); project administration (supporting); resources (equal); software (equal); supervision (lead); validation (equal); visualization (equal); writing – original draft (supporting); writing – review and editing (equal). **Loth S. Mulungu:** Conceptualization (equal); data curation (supporting); formal analysis (equal); funding acquisition (supporting); investigation (equal); methodology (equal); project administration (supporting); resources (equal); software (equal); supervision (lead); validation (equal); visualization (equal); writing – original draft (supporting); writing – review and editing (equal).

## CONFLICT OF INTEREST

Authors declare no conflict of interest among themselves.

## Data Availability

Authors agree to deposit the data associated with this study in an Institutional repository of Sokoine University of Agriculture SUA and make it publicly available, once the manuscript is accepted for publication under the Journal of Ecology and Evolution. https://www.suaire.sua.ac.tz/handle/123456789/4206.

## APPENDIX A Pearson's correlation matrix indicating correlation coefficients (*r* ≥ .5) for independent variables

Canopy cover CCv was highly correlated with herbs density. Altitude was correlated with BD bulk density and MST soil moisture. Ground cover GCv was correlated with shrubs density and MST soil moisture. Multiple correlated variables such as canopy cover, soil moisture and altitude were not included in the models.

## APPENDIX B Model selection results of the 15 models based on the AIC

**Table:**

Model	Details	df	AIC	ΔAIC
1	Richness ~ 1	2	6029.05	769.73
2	Richness ~ Soil type	5	6029.51	770.19
3	Richness ~ Habitat	8	5549.76	290.44
4	Richness ~ Season	3	6030.88	771.57
5	Richness ~ GCv	3	5501.59	242.28
6	Richness ~ Habitat + Season	9	5551.53	292.21
7	Richness ~ Habitat + GCv	9	5312.27	52.95
8	Richness ~ GCv + Herbs	4	5489.96	230.64
9	Richness ~ Habitat + Season + GCv	10	5313.95	54.64
**10**	**Richness ~ Habitat + Season + GCv + Soil type**	**13**	**5259.31**	**0.00**
11	Richness ~ Habitat +Season + GCv + Soil type + BD	14	5260.42	1.11
12	Richness ~ Habitat + Season + GCv + Soil type + BD + Herbs	14	5260.73	1.42
13	Richness ~ Habitat + Season + Habitat*Season + GCv + Soil type	19	5263.10	3.79
14	Richness ~ Habitat + Season + Habitat*Season + GCv + Soil type + BD + Herbs	20	5264.22	4.91
15	Richness~ Habitat + Season + Habitat*Season + GCv	16	5318.19	58.87

Abbreviations: GCv, ground cover; BD, bulk density; Herbs, herb density.

Bold indicates: significant at 0***.

## APPENDIX C Model selection results of the 15 models based on the AIC

**Table:**

Model	Details	df	AIC	Δ AIC
1	Abundance ~ 1	2	9201.28	538.04
2	Abundance ~ Soil type	5	9201.83	538.58
3	Abundance ~ Habitat	8	8804.52	141.28
4	Abundance ~ Season	3	9201.79	538.55
5	Abundance ~ GCv	3	8869.89	206.65
6	Abundance ~ Habitat + Season	9	8800.75	137.51
7	Abundance ~ Habitat + GCv	9	8716.94	53.69
8	Abundance ~ GCv + Herbs	4	8865.80	202.56
9	Abundance~ Habitat + Season + GCv	10	8714.52	51.27
10	Abundance ~ Habitat + Season + GCv + Soil type	13	8664.87	1.63
**11**	**Abundance ~ Habitat + Season + GCv + Soil type + BD**	**14**	**8663.24**	**0.00**
12	Abundance ~ Habitat + Season + GCv + Soil type + BD + Herbs	14	8667.82	4.58
13	Abundance ~ Habitat + Season + Habitat*Season + GCv + Soil type	19	8669.38	6.14
14	Abundance ~ Habitat + Season + Habitat*Season + GCv + Soil type + BD + Herbs	20	8669.05	5.81
15	Abundance ~ Habitat + Season + Habitat*Season + GCv	16	8717.81	54.57

*Note*: Model with the lowest AIC (shown in bold) is the one that better describes and fits our data. For every model; the number of parameters (df), AIC and delta ΔAIC are given. ΔAIC is the difference in AIC between the current model and the best model.

Abbreviations: GCv, ground cover; BD, bulk density; Herbs, herb density.

Bold indicates: significant at 0***.
