# Corrigendum

**DOI:** 10.1002/ece3.9403

**Published:** 2022-10-03

In the recent article by Thomas et al. (2022), the authors would like to correct the article title. The corrected article title is shown below and has been applied to the online version:

Influence of vegetation structure, seasonality, and soil properties on rodent diversity and community assemblages in west Mount Kilimanjaro, Tanzania

The authors apologize for the error.
